# FPIES: Reviewing the Management of Food Protein-Induced Enterocolitis Syndrome

**DOI:** 10.1155/2016/1621827

**Published:** 2016-03-08

**Authors:** Neha Khanna, Kirtika Patel

**Affiliations:** ^1^Lister Hospital, East and North Hertfordshire NHS Trust, Stevenage SG1 4AB, UK; ^2^Department of Immunology, School of Medicine, Moi University, P.O. Box 4606, Eldoret 30100, Kenya

## Abstract

*Purpose of Review.* The aim of this review is to provide a case driven presentation of the presenting features and diagnostic criteria particularly focusing on the management of FPIES. It also summarises the natural history and resolution of cow's milk induced FPIES.* Data Sources.* OvidSP Database was used to search for literature using the keywords food protein-induced enterocolitis and FPIES.* Recent Findings.* The diagnosis of FPIES is often delayed following two or more presentations. Symptoms in the acute form include profuse vomiting usually 2–6 hours following ingestion of food. Vomiting may or may not be accompanied by diarrhoea. Management involves removing the causal food protein from diet. There is some concomitance in cow's milk and soya induced FPIES. Hence extensively hydrolysed formula is the milk of choice unless breast-feeding is carried out in which case that should be continued.* Summary.* FPIES is a complex form of non-IgE mediated food allergy. More awareness and knowledge of the condition are required to prevent misdiagnosis. Early diagnosis and removal of the culprit food protein improve the outcome. Good nutritional advice and clear management plans are important. More multicentre studies are required to reevaluate and produce consistent oral food challenge criteria and guidelines.

## 1. Background

FPIES is a rare non-IgE mediated food allergy triggered by the ingestion of certain food proteins. The symptoms are solely gastrointestinal, vomiting with or without diarrhoea leading to hypotension and lethargy [1]. The pathophysiological mechanisms are not yet fully understood. The incidence of FPIES is difficult to ascertain because of frequent misdiagnosis. However a large birth cohort study conducted in Israel reported an incidence of 0.34% for FPIES to cow's milk [1].

FPIES is diagnosed on the basis of history and typical symptoms that improve with avoidance of the culprit food. Oral food challenge (OFC) remains the gold standard of diagnosis [2].

## 2. Case History Presentation

This is a case history of a two-year-old boy who presented to a Paediatric Allergist at six months of age following three episodes of severe reaction to cow's milk at various stages.

The infant was born by normal delivery following an in vitro fertilization pregnancy and weighing 3.2 kilograms. He was exclusively breast-fed since birth with the mother consuming a normal diet inclusive of dairy, soy, and egg. The infant first direct exposure to cow's milk was at the age of four months when he was fed a cow's milk formula feed. Two hours following this feed, he developed profuse vomiting, which lasted for an hour. He then developed lethargy and an unusual deep sleep for several hours following this episode and it was difficult for him to rouse from sleep. The episode resolved after he had slept for a long time. He was managed at home by parents and taken to primary care practitioner the following day.

This reaction was reproduced again at the age of five months when he was fed a different cow's milk formula feed. At the age of six months, when given a baby porridge containing cow's milk, a similar reaction was noticed. There was no associated diarrhoea with either of the episodes and stools were otherwise normal.

He had been weaned onto solids at the age of six months and tolerated various products such as eggs, fish, wheat, rice, oats, beans, lentils, poultry, and sweet potato without any problems. He did not have any symptoms suggestive of gastroesophageal reflux.

The infant also developed eczema at the age of three months, which was initially treated with mild topical corticosteroids followed by a brief period of usage of potent corticosteroids. He did not have any signs of asthma, allergic rhinitis, or any other allergies. Mother had eczema and an immediate egg allergy as a child. Father had eczema when he was young.

A diagnosis of FPIES to cow's milk protein was made on the basis of history, clinical examination, and skin prick testing as shown in Table 1. Prior to this presentation to the allergy service, parents had sought medical attention (primary care) after each episode of reacting to cow's milk and had been reassured that this was not an allergic reaction to cow's milk. They were advised to try a different brand of cow's milk formula each time.

Parents were thereafter advised to avoid any form of cow's milk products in his diet. Mother was advised to continue breast-feeding if she wished to without the need to exclude dairy in her diet. At the age of nine months, he was commenced on an extensively hydrolysed formula milk as mother wanted to discontinue breast-feeding.

At the age of twelve months, he had an OFC to soy milk in hospital, which he passed. He was then transitioned from EHF to soy milk.

At the age of 2 years and 6 months, the child was challenged with fresh cow's milk. This was done as a high-risk OFC in hospital with an intravenous cannula placed before the beginning of the procedure. The protocol used for OFC to cow's milk in hospital to check if tolerance had developed is elaborated as follows [3].

He was given cow's milk in two stages:

*Dose 1: 4.4 mls/kg Cow's Milk (Protein Content 0.15 grams/kg)*

 Observe the child for 45 minutes and do a full set of observations (blood pressure, respiratory rate, pulse, oxygen saturations, PEFR (if applicable), and chest auscultation) and clinical assessment every 15 minutes. If there was no reaction after 45 minutes, proceed to the next stage.

*Dose 2: 4.4 mls/kg Cow's Milk (Total Cumulative Protein Content 0.3 grams/kg)*

 Observe the child for 4 hours. For the initial hour do a full set of observations (blood pressure, respiratory rate, pulse, oxygen saturations, PEFR (if applicable), and chest auscultation) and clinical assessment every 15 minutes. For the remaining 3 hours do a full set of observations and clinical assessment every 30 minutes.

## 3. Discussion

### 3.1. Pathophysiology

The underlying pathophysiology is not well understood and is thought to be a cell mediated food hypersensitivity [4].

Current understanding is that ingestion of food allergens causes T cell activation and local inflammation and hence leads to increased intestinal permeability and rapid fluid shifts and causing typical symptoms of FPIES [5].

There is some suggestion that this inflammation may be mediated by TNF-*α*, and a relative lack of expression of TGF-*β* may be involved, causing increased mucosal permeability and leading to rapid fluid shifts [4, 6].

Leucocytosis with left shift is also a common finding in patients with acute FPIES [7, 8].

### 3.2. Clinical Features and Presentation

There are two types of FPIES described in literature, acute and chronic [9, 10]. The acute form presents typically 2–6 hours after ingestion of the culprit food, and hence the delay in symptoms often leads to misdiagnosis as either sepsis or acute gastroenteritis [9]. The chronic form, which is less common, is attributed to the constant exposure to the triggering food. The chronic form presents in a subacute form with vomiting, diarrhoea, faltering growth, and dehydration [9, 10]. This makes it difficult to diagnose as the presentation overlaps with other possible gastrointestinal conditions.

### 3.3. Offending Foods

The most common offending foods are cow's milk followed by soya [6, 11].

A large retrospective study in America showed cow's milk (67%) as the most common trigger for FPIES followed by soya (41%) and then followed by grains (34.6%) and egg (11%) [11]. Another recent study in USA showed that the most common foods were cow's milk (44%), soy (41%), rice (22.5%), and oat (16%). The majority (65%) reacted to 1 food, 26% reacted to 2 foods, and 9% reacted to 3 or more foods [12].

Apart from cow's milk and soy, other foods reported to cause FPIES are rice, grains (oat, barley, and wheat), poultry (chicken and turkey), legumes (green peas, lentils, string beans, and peanut), sweet potato, squash, egg white, fruit, white potato, lamb, and fish [2, 13].

### 3.4. Diagnosis

FPIES is a clinical diagnosis [7]. Diagnosis of FPIES is made on the basis of history and clinical symptoms and by excluding other causes. Allergy tests including skin prick tests and specific IgEs are mostly negative [1, 6, 13] but can be positive in up to 30% patients having positive tests [1, 6, 13]. These patients have a possibility of developing IgE mediated cow's milk allergy [1]. OFC is the gold standard of diagnosis but often not needed if the history is classic and removal of the offending food resolves symptoms [14].

Powell's criteria [7] are the most widely used for diagnosis and include the following:

1. Less than 9 months old at initial reaction.
2. Exposure to the culprit food resulting in profuse vomiting and lethargy and/or diarrhoea within 4 hours.
3. Symptoms limited to the gastrointestinal tract.
4. Elimination of the culprit food from the diet resulting in resolution of symptoms.
5. An OFC or isolated reintroduction of the culprit food eliciting the typical symptoms.

Newly propped criteria have been suggested in a recent review and are listed below [15]:

*Major Criteria*

1. Repetitive vomiting or diarrhoea within 6 hours of food ingestion.
2. Absence of cutaneous and respiratory symptoms suggestive of an IgE mediated allergy.
3. Removal of causative food which results in resolution of symptoms.
4. Reexposure or a food challenge eliciting the typical symptoms.

*Minor Criteria*

- Hypotension.
- Lethargy, pallor, or hypotonia.
- Negative skin prick test and undetectable specific IgE level.
- Absence of fever or hypothermia (less than 36 degrees Celsius).

The child in this case had a delay in diagnosis of two months and had multiple presentations to the primary care physician before an allergist finally saw him. This observation is in keeping with the findings of a large UK retrospective study [9], which showed that patients had a mean delay of 12 months in the diagnosis of FPIES and had a mean of at least 2 presentations to a medical professional before allergy or gastroenterology input was received. The patients presented with vomiting, diarrhoea, and signs suggestive of hypotension and vomiting was always more prominent than diarrhoea [9].

### 3.5. Management

Management of acute reactions in FPIES involves treating the episode once recognized. Most episodes will resolve with fluid rehydration and may even be self-resolving [1, 4]. Adrenaline is useful only in IgE mediated reactions and hence does not have a role in management of FPIES unless there is hypotension not responsive to fluid resuscitation [16–19].

Steroids (single dose of methylprednisolone) are recommended in cases with more severe symptoms [15, 16].

Ondansetron has been shown to be effective in the resolution of symptoms in acute FPIES in some studies on a small number of patients during OFC. A complete resolution of symptoms was within 10–15 mins of administering ondansetron (intravenous or intramuscular) [20, 21].

Long-term dietary management consists of removing the offending food from the diet with careful advice and use of action plans [17].

#### 3.5.1. Maternal Exclusion Diet and Role of Breast-Feeding

It is well reported that most infants with acute FPIES will tolerate breast milk from an unrestricted maternal diet. Two large studies have reported that infants with FPIES did not seem to react to allergens in breast milk [13, 22]. However there are case reports where infants have reacted to the allergen in maternal breast milk. But as these are rare, most studies recommend having a more tolerant approach unless infant remained symptomatic or is failing to thrive when breast-feeding [23, 24]. Hence, if the infant's mother was consuming the offending food during breast-feeding and the infant did not present with FPIES, the mother is not routinely advised to avoid the same food in her diet [17].

As the diagnosis of chronic FPIES may overlap with other gastrointestinal conditions, no clear link has been established between the symptoms and maternal milk intake when breast-feeding. However if the child is failing to thrive, a trial of exclusion diet for mother under the guidance of a dietician may be recommended [17].

#### 3.5.2. Choice of Formula Milk for Non-Breast-Feeding Babies

Some large studies from Australia [8], Israel [1], and Italy [4] show that there was no concomitance in cow's milk and soy induced FPIES. There is however a discrepancy in literature regarding this and some studies from USA and Korea show that there are a large proportion of infants who have concomitant FPIES to cow's milk and soya [13, 25]. EHF is recommended in the management of FPIES to infants who cannot be breast-fed [6, 18, 26].

The Australian and American consensus guidelines also recommend EHF for treatment of FPIES [22, 27, 28].

The choice of formula recommended for IgE mediated cow's milk allergy is EHF [29].

#### 3.5.3. Weaning

The usual age for presentation of FPIES is between 4 and 6 months [8]. The usual weaning foods tend to be baby rice, oats, corn based porridge, fruits, and vegetables followed by yogurt and fromage frais (soft white cheese that has the consistency of yogurt) [30]. The fact that FPIES can occur to atypical allergenic foods like rice, oats, and chicken can complicate the weaning process [14]. In a child with FPIES, weaning guidance has been suggested in reviews where foods unlikely to cause FPIES are recommended to be tried first but clinical judgement should be used in each case. Warning the patients of possibility of multiple triggers and good dietetic advice is essential to support through the weaning process [31].

#### 3.5.4. When Should an OFC Be Performed to Test Tolerance Achievement?

OFC can be used either to establish the diagnosis or to test the achievement of tolerance. However, most clinicians and parents would not be willing to challenge for diagnosis confirmation as the child has usually had a few serious episodes by the time the diagnosis is made.

Various studies have looked at the age of challenging for achievement of tolerance. For cow's milk, the age at which tolerance was regained is somewhere in the second year of life. One study reported that 60% of children had developed tolerance to cow's milk at age of 3 years [13]. Another study found that most of their patients had achieved tolerance at age of 18–20 months [25]. Another Italian study reported resolution of cow's milk FPIES at age of 18–24 months [4].

#### 3.5.5. Oral Food Challenge Guidelines

FPIES is a high-risk challenge and should be performed in a setting where the resuscitation facilities are present. This would mostly be an in-patient setting. There are several protocols documented for performing an FPIES challenge. The protocol described by Leonard and Nowak-Węgrzyn [32] comprises administering the offending food in 3 portions during 45–60 minutes for a total of 0.06 to 0.6 grams/kg of food protein starting at a lower dose in patients with a history of severe reactions. If the patient remains asymptomatic after 2 to 3 hours, then an age appropriate serving is usually given as a second serving and the patient is observed for several hours. The Italian study [4] highlighted that the reactions always occurred after a whole meal had been eaten (200 mls cow's milk, 100 grams fish, or a whole egg). Hence finding the trigger dose to elicit an FPIES reaction is difficult as the symptoms are always delayed.

In this case, the local protocol was used to do an OFC which has been written as per guidance by Powell [7] and updated by Sicherer and colleagues [6, 18].

#### 3.5.6. Prognosis

Prognosis for FPIES is favourable with overall 85% patients having resolution of their reactions by 5 years of age [11]. However this is population dependent and different studies have shown varying resolution rates. One study showed that 60% of cow's milk sensitive patients showed resolution by 2 years of age [6]. However, only 25% of soya sensitive patients symptoms resolved by 2 years [6]. In another previous study, 80% had lost their reactivity to soya by three years of age [8]. A recent American study has shown that the median age for tolerance to be established was 5.1 years in children with undetectable IgE levels to cow's milk. The median age of resolution for soy was 6.7 years, rice was 4.7 years, and oat was 4 years [12].

Positivity for food specific IgE remains a poor prognostic marker for outgrowing the hypersensitivity [6, 12, 13].

## 4. Conclusion

To summarise, “FPIES is not an apple pie” and diagnosis and management can be quite challenging. It is a rare condition and is often underrecognized. The patient has usually had multiple presentations before being diagnosed. Diagnosis is clinical with allergy tests often being negative. Once recognized, management involves removing the causal food protein from the diet and this results in dramatic improvement. Breast-feeding is mostly well tolerated and most patients do not react to the food protein in breast milk. The first choice of formula milk for non-breast-feeding infants is EHF. OFCs are important in determining if tolerance to the offending food has developed. For cow's milk, OFC can be performed after the age of 18 months. This involves giving the food in 2-3 stages with the last stage being an age appropriate serving followed by a period of observation for several hours. Prognosis is usually good but age of resolution varies with populations with a small percentage developing IgE mediated food allergy.

## Figures and Tables

**Table 1:** Skin prick testing at six months of age.

Allergen being tested	Size of wheal (in millimetres)	Allergen being tested	Size of wheal (in millimetres)
Positive control (histamine)	3	Negative control (normal saline)	0
Cow's milk	0	Egg	0
Soy	0	Wheat	0
Shrimp	0	Peanut	0
Hazelnut	0	Sesame	0
Cashew nut	0		

## Abbreviations

FPIES: Food protein-induced enterocolitis syndrome
OFC: Oral food challenge
EHF: Extensively hydrolysed formula
TNF-*α*: Tumour necrosis factor-alpha
TGF-*β*: Transforming growth factor-beta
mls: Millilitres.
